# Microcalcifications in breast cancer: novel insights into the molecular mechanism and functional consequence of mammary mineralisation

**DOI:** 10.1038/bjc.2011.583

**Published:** 2012-01-10

**Authors:** R F Cox, A Hernandez-Santana, S Ramdass, G McMahon, J H Harmey, M P Morgan

**Affiliations:** 1 Molecular and Cellular Therapeutics, Royal College of Surgeons in Ireland, 123 St Stephen's Green, Dublin 2, Ireland; 2 School of Chemical Sciences, Dublin City University, Dublin 9, Ireland

**Keywords:** microcalcifications, breast cancer, mineralisation, calcifications, hydroxyapatite, mammography

## Abstract

**Background:**

Mammographic microcalcifications represent one of the most reliable features of nonpalpable breast cancer yet remain largely unexplored and poorly understood.

**Methods:**

We report a novel model to investigate the *in vitro* mineralisation potential of a panel of mammary cell lines. Primary mammary tumours were produced by implanting tumourigenic cells into the mammary fat pads of female BALB/c mice.

**Results:**

Hydroxyapatite (HA) was deposited only by the tumourigenic cell lines, indicating mineralisation potential may be associated with cell phenotype in this *in vitro* model. We propose a mechanism for mammary mineralisation, which suggests that the balance between enhancers and inhibitors of physiological mineralisation are disrupted. Inhibition of alkaline phosphatase and phosphate transport prevented mineralisation, demonstrating that mineralisation is an active cell-mediated process. Hydroxyapatite was found to enhance *in vitro* tumour cell migration, while calcium oxalate had no effect, highlighting potential consequences of calcium deposition. In addition, HA was also deposited in primary mammary tumours produced by implanting the tumourigenic cells into the mammary fat pads of female BALB/c mice.

**Conclusion:**

This work indicates that formation of mammary HA is a cell-specific regulated process, which creates an osteomimetic niche potentially enhancing breast tumour progression. Our findings point to the cells mineralisation potential and the microenvironment regulating it, as a significant feature of breast tumour development.

The appearance of mammographic mammary microcalcifications is routinely used to detect breast cancer in its early stages allowing more conservative therapy and better patient outcomes. Up to 50% of all nonpalpable breast cancers are detected solely through microcalcifications presenting during a mammogram scan (Gulsun *et al*, 2003) and up to 93% of cases of ductal carcinoma *in situ* (DCIS) present with microcalcifications (Hofvind *et al*, 2011). There is mounting evidence to suggest that mammary microcalcifications are associated with a poorer prognosis for certain breast cancer patients. Studies have shown that breast cancers presenting with microcalcifications are more often associated with lymph node invasion (Holme *et al*, 1993) and HER-2 positivity (Seo *et al*, 2006; Wang *et al*, 2008). A study by Tabar *et al* (2004) has investigated whether mammographic features of microcalcifications can be used to reliably predict the long-term outcome for women with small (1–14 mm) invasive breast cancers. A subgroup of women within the study who presented with mammographic casting-type calcifications was found to have unexpectedly poor survival rates for this tumour-size category. Although this finding has been the subject of some debate in the literature (James *et al*, 2003; Evans *et al*, 2006; Mansson *et al*, 2009), other research groups have reported similar findings that support the work of Tabar *et al* (Thurfjell *et al*, 2001; Peacock *et al*, 2004).

Mammary microcalcifications have been classified at a molecular level and are distinguished by their physical and chemical properties. Type I calcifications are composed of calcium oxalate (CO), which are amber in colour, partially transparent and form pyramidal structures with relatively planar surfaces. Type II calcifications are composed of calcium phosphate, mainly hydroxyapatite (HA), which are grey-white, opaque and form ovoid or fusiform shapes with irregular surfaces (Frappart *et al*, 1984). Calcium oxalate has been shown to be associated with benign lesions of the breast or at most non-invasive lobular carcinoma *in situ*, whereas HA is associated with both benign and malignant breast conditions (Büsing *et al*, 1981; Frappart *et al*, 1984, 1986; Radi, 1989; Haka *et al*, 2002). It has been suggested that HA calcifications formed in benign ducts contain smaller amounts of protein and higher amounts of calcium carbonate than those formed in malignant ducts (Haka *et al*, 2002). A more recent study supports this concept and has also shown that the carbonate content of HA decreases with increasing lesion grades (Baker *et al*, 2010).

Little research has been carried out to investigate the impact that the presence of HA has within the tumour microenvironment, however, this group has previously reported on the biological effects of exogenous HA on mammary cell lines. We have previously shown that HA enhances the mitogenesis of mammary cells, which may amplify the malignant process by aggravating tumour growth (Morgan *et al*, 2001). Increased expression of prostaglandin E_2_ was also found to be associated with HA treatment (Morgan *et al*, 2001) and elevated levels of prostaglandins are known to be a marker of high metastatic potential (Rolland *et al*, 1980). The inflammatory cytokine IL-I*β* was also found to be upregulated in mammary cell lines in response to exogenous HA, which could promote a pro-inflammatory microenvironment (Cooke *et al*, 2003). HA was also shown to upregulate a variety of matrix metalloproteinases (MMPs), which are known to degrade the basement membrane allowing cancer cells to invade into surrounding tissues (Morgan *et al*, 2001).

Despite the importance of mammary microcalcifications for the early detection of breast cancer and the potential prognostic and biological relevance, little research has been carried out to investigate the molecular mechanisms involved in their formation. In the past, mammary calcification has been considered a passive, end stage process associated with cellular degeneration with no tangible biological significance. However, studies in other cellular systems have suggested that the mechanisms regulating pathological mineralisation may be similar to those involved in physiological mineralisation of bone (Giachelli, 2004; Kirsch, 2006; Shroff and Shanahan, 2007), which is widely considered to be an active regulated process (Stein and Lian, 1993; Giachelli, 2005; Orimo, 2010). Breast cancer biopsies have been shown to overexpress several bone matrix proteins, including bone sialoprotein, osteopontin (OPN) and osteonectin. In addition, their expression is associated with frequent microcalcification deposition in the breast lesions (Bellahcene *et al*, 1994; Bellahcene and Castronovo, 1995). To date, no *in vitro* models of mammary cell mineralisation exist to study the molecular mechanisms involved in this process. The aim of this study was to establish and characterise a reproducible *in vitro* model of mammary cell mineralisation, from which the molecular mechanisms underlying mammary mineralisation can begin to be elucidated.

## Materials and methods

### Cell lines and media

The murine mammary adenocarcinoma 4T1 cell line was generously provided by Dr Fred Miller (Duke University, NC, USA) (Aslakson and Miller, 1992). The murine mammary adenocarcinoma 4T1.2 cells that preferentially metastasis to bone, were a gift from Robin Anderson (Peter MacCallum Cancer Centre, Australia) (Lelekakis *et al*, 1999). The 4T1 and 4T1.2 cell lines were maintained in low glucose DMEM, 10% FBS and 1% penicillin/streptomycin. The MCF10a, Hs578T and Hs578Ts(i)_8_ cell lines were a gift from Dr Susan McDonnell (University College Dublin, Ireland). The MCF10a cells were maintained in DMEM/F-12, 5% horse serum, 0.5 *μ*g ml^–1^ hydrocortisone, 100 ng ml^–1^ cholera toxin, 0.2 mg ml^–1^ epidermal growth factor, 10 *μ*g ml^–1^ bovine insulin and 1% penicillin/streptomycin. The Hs578T and Hs578Ts(i)_8_ cells (Hughes *et al*, 2008) were maintained in high glucose DMEM, 10% FBS, 10 *μ*g ml^–1^ bovine insulin and 1% penicillin/streptomycin. All cell culture reagents were purchased from Sigma-Aldrich (Arklow, Ireland) and Biosera (East Sussex, UK).

### Assessment of mineralisation *in vitro*

4T1, 4T1.2 and MCF10a cells were seeded into six-well culture plates (day −1) at 1.5 × 10^5^ cells per well and Hs578T and Hs578Ts(i)_8_ cells were seeded at 7.5 × 10^4^ cells per well. The following day (day 0) the cells were treated with regular growth media or an osteogenic cocktail (OC) (50 *μ*g ml^–1^ ascorbic acid, 10 mM *β*-glycerophosphate (*β*G) ±10^−7^ M dexamethasone). The effects of additional exogenous factors were also investigated including inorganic phosphate (Pi) (10 mM; Sigma-Aldrich, #30427 and #71496), phosphonoformic acid (PFA) (1 mM; Sigma-Aldrich, #79510), levamisole (100 *μ*M; Sigma-Aldrich, #L9756), alkaline phosphatase (ALP) (1 U ml^–1^; Sigma-Aldrich, #79390), OPN (0.5 *μ*g ml^–1^; R&D Systems Europe Ltd., Abingdon, UK, #109-OP) and inorganic pyrophosphate (PPi) (3.5 *μ*M; Sigma-Aldrich, #P8010). Cells were grown for up to 28 days in a humidified incubator at 37 °C and 5% CO_2_. Mineralisation was assessed using alizarin red S staining, von Kossa staining, quantitative calcium assay and Raman microspectroscopy, as described below.

### Assessment of mineralisation *in vivo*

All mice were housed in a licensed biomedical facility (RCSI, Beaumont Hospital) and had *ad libitum* access to food and water. The animals were caged in groups of 5 or less and were acclimatised to their environment for 1 week. Cages were kept in an air-conditioned room (21–22 °C) and were on a 12-h light–dark cycle. All procedures were subjected to institutional ethics review and were carried out under the animal license guidelines of the Department of Health and Children, Ireland and in accordance with the UK Co-ordinating Committee on Cancer Research guidelines for the welfare of animals in experimental neoplasia (1998). 4T1.2 or 4T1 cells (5 × 10^4^) were implanted into the mammary fat pad of 10- to 12-week-old female BALB/c mice. Mice were killed when tumours reached a mean tumour diameter (square root of the product of length by breadth) of 17 mm. Tumours were excised, fixed in 10% paraformaldehyde and embedded in paraffin wax. Mineralisation was assessed using alizarin red S and von Kossa staining, as described below.

### Histological staining

Cell monolayers were fixed with 10% formalin for 30 min and stained with alizarin red S (2%, pH 4.4) for 4 min. For von Kossa staining, silver nitrate (5%) was applied for 1 h under an electric lamp followed by sodium thiosulphate (5%) treatment for 2 min. For paraffin-embedded tissue, serial sections (10 *μ*m) were deparaffinised using xylene and rehydrated before staining with alizarin red S, von Kossa (using nuclear fast red counterstain) or haematoxylin and eosin (H&E). Samples were then dehydrated, incubated with xylene (30 min) and mounted with DPX.

### Quantitative o-cresolphthalein calcium assay

Calcium was extracted from cell monolayers by incubation with nitric acid (1 M) for 1 h. The absorbance of the samples (70 *μ*l) in combination with o-cresolphthalein (0.1 mg ml^–1^; 70 *μ*l) and 2-amino-2-methyl-1-propanol (90 mg ml^–1^; 175 *μ*l) was read at 572 nm (Yavorskyy *et al*, 2010). A protein assay was carried out on duplicate wells. Cells were harvested in RIPA lysis buffer (1 × PBS; 1% NP-40, 0.5% sodium deoxycholate, 0.1% SDS) containing 1% protease inhibitor cocktail (Sigma-Aldrich, #P8340), stored on ice for 1 h with occasional vortexing and centrifuged at 12 000 r.p.m. for 20 min. The supernatant was used for the BCA protein assay (Novagen, Merck Group, Darmstadt, Germany), which was carried out according to the manufacturer's instructions.

### Raman microspectroscopy

Cell monolayers were fixed with 100% methanol for 30 min. Raman spectra were acquired on a Witec Alpha 300 R microscope (Ulm, Germany) equipped with a 532 nm Ar-Ion laser (∼40 mW) with a spectral resolution of 3 cm^−1^ using backscattering configuration and a × 100 objective (NA=0.9). Point spectra were acquired from areas of mineral deposition initially identified visually using an optical image. Spectra were processed using Witec Project v2.0 60 software. Measurements were taken from five different points for each well, including biological repeats.

### Real-time RT–PCR

RNA was extracted using trizol (Invitrogen, Life Technologies, Carlsbad, CA, USA, #15596) and reverse transcribed using a high-capacity cDNA reverse transcription kit (Applied Biosystems, Life Technologies, #4368814). Real-time PCR was carried out using the real-time PCR thermocycler (Applied Biosystems, 7500) for ALP (Qiagen, West Sussex, UK, QT00157717), OPN (Qiagen, QT00157724) and 18s (Qiagen, QT01036875) as an endogenous control. Samples were heated at 95 °C for 15 min, followed by a second stage composed of 15 s at 94 °C, 30 s at 55 °C and 45 s at 72 °C, which was repeated 40 times.

### Detection of ALP activity

Cells were harvested in a buffer (1% Triton X-100, 0.9% NaCl) and kept on ice for 1 h with occasional vortexing. Samples were centrifuged at 12 000 r.p.m. for 20 min and the supernatant was used for the ALP assay. Samples (50 *μ*l) and p-nitrophenyl phosphate (100 *μ*l) were incubated at 37 °C for 30 min and absorbance was measured at 405 nm. The results were normalised to protein content in the samples using the BCA protein assay (Novagen), which was carried out according to the manufacturer's instructions.

An ALP stain was also used. Cell monolayers were fixed using 100% methanol for 30 min. Tris-HCl (pH 8.5) containing naphthol AS-MX phosphate (1 mg ml^–1^) and fast red violet LB (0.6 mg ml^–1^) was added to each well, which were incubated at 37 °C for 45 min. Images were recorded at × 100 magnification using the Nikon eclipse TS100 inverted light microscope (Nikon, Surrey, UK).

### Assessing migration by scratch wound assay

4T1 cells were seeded into six-well plates (7 × 10^5^ cells per well) and the following day multiple scratch wounds were created in the monolayers. In the presence of 0.5% FBS media, the following treatments were added; 1.8–10 mM calcium, 0–10 mM *β*G, 0–72 *μ*g cm^–2^ CO monohydrate (Sigma-Aldrich, #21201) or 0–72 *μ*g cm^–2^ HA (Sigma-Aldrich, #677418). The HA and CO crystals were sterilised by heating at 200 °C for 90 min and sonicated briefly before their use. The cells were kept in a humidified incubator at 37 °C containing 5% CO_2_. Tiff images of the scratch wound assays taken at × 100 magnification at 0, 24 and 48 h were imported into Scion Image software (Scion Corporation Ltd, Frederick, MD, USA), which is used to generate wound size measurements from still images (McSherry *et al*, 2011). The mean closure of the wound was calculated from six individual measurements for each wound at each time point. This process was carried out for all biological repeats. The day 0 scratches were designated as 100% open. From this, the % closure for all scratches was calculated. The % closure represents the distance that the cells migrated into the scratch wound area over time. Results are presented as mean % closure ±s.e.m.

### Statistical analysis

All statistical analysis was carried out using GraphPad Prism 5 software (La Jolla, CA, USA). A one-way analysis of variance (ANOVA) was used to analyse multiple treatment groups. A two-way ANOVA was used to analyse multiple treatments over multiple time points. *Post-hoc* analysis was carried out when statistical significance (*P*<0.05) was detected.

## Results

### *In vitro* mineralisation of metastatic 4T1 mammary adenocarcinoma cells

**Figure 1:**
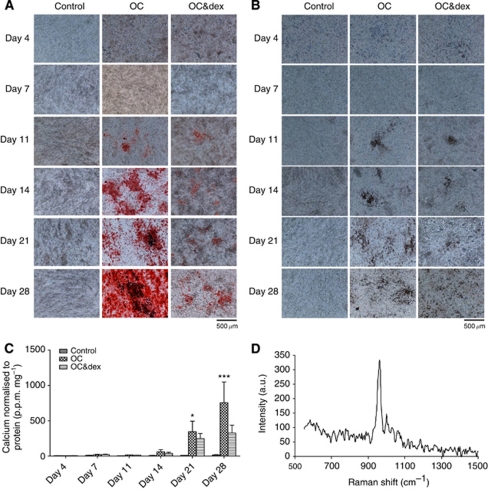
Investigating the mineralisation potential of 4T1 cells. Representative Alizarin red S (**A**) and von Kossa (**B**) staining of 4T1 cells over time in culture (original magnification × 100) (*n*=3). Scale bars represent 500 *μ*m. (**C**) The calcium content of 4T1 cells as determined by the o-cresolphthalein calcium assay and normalised to protein. Each point represents the mean amount of calcium measured in p.p.m. normalised to protein measured in mg, ±s.e.m., two-way ANOVA. ^*^P<0.05 OC *vs* control on day 21. ^***^P<0.001 OC *vs* control on day 28. (**D**) Raman spectroscopy of mineralising 4T1 cells grown in the OC for 28 days showing a peak at 960 cm^−1^. Control=regular growth media. Osteogenic cocktail=regular growth media supplemented with 50 *μ*g ml^–1^ ascorbic acid and 10 mM *β*G. OC&dex=regular growth media supplemented with 50 *μ*g ml^–1^ ascorbic acid, 10 mM *β*G and 10^−7^ M dexamethasone.

In order to study the molecular mechanisms of mammary mineralisation, it was necessary to first establish and characterise a reproducible *in vitro* model. The highly metastatic mouse mammary 4T1 cell line was grown in culture plates for up to 28 days in the presence of regular growth media (control), an OC (10 mM *β*G and 50 *μ*g ml^–1^ ascorbic acid in regular growth media) or the OC containing 10^−7^ M dexamethasone (OC&dex). Cell monolayers were stained with alizarin red S and von Kossa to assess mineralisation and observed under the light microscope at × 100 magnification. Positive staining was detected in both the OC and OC&dex-treated groups using alizarin red S (red for calcium) and von Kossa staining (black/brown for calcium phosphate), beginning on day 11 (Figures 1A and B). This staining increased in intensity over time with the strongest staining observed in the OC group. These findings were confirmed by quantitative o-cresolphthalein calcium assay (Figure 1C). A statistically significant increase in calcium was detected on days 21 (*P*<0.05) and 28 (*P*<0.001) in the OC group compared with the control group. Although an increase in calcium was also observed in the OC&dex group over time, this was not found to be statistically significant (*P*>0.05). Raman microspectroscopy was used to identify the mineral species being deposited (Figure 1D). The Raman band observed at 960 cm^−1^ in mineralising 4T1 samples corresponds to a symmetric phosphate stretching mode, which is indicative of HA (Nelson and Williamson, 1982).

### Mineralisation potential of mammary cell lines is associated with phenotype

The mineralisation potential of several additional murine and human mammary cell lines was also investigated. These results are summarised on Table 1. In addition to the 4T1 cell line, a highly metastatic mammary subclone designated 4T1.2, which was originally isolated from the parental 4T1 cells (Lelekakis *et al*, 1999), also produced HA *in vitro*. This occurred in response to both the OC and OC&dex by day 14. Three human mammary cell lines were also examined. Normal immortalised MCF10a cells and the Hs578T cell line were not capable of mineralising under the same experimental conditions. However, a more invasive subclone designated Hs578Ts(i)_8_ (Hughes *et al*, 2008) produced HA after 21 days growth with OC&dex treatment. The Raman band at ∼960 cm^−1^ detected in all mineralising samples is typical of HA (Nelson and Williamson, 1982). The full width at half height (FWHH) of the Raman phosphate peak was also measured, as previous studies have shown that this reflects the carbonate content of HA (Haka *et al*, 2002). The FWHH is narrower for the Hs578Ts(i)_8_ mineralising samples (15.85±1.7 cm^−1^) compared with that found in the 4T1 (23.89±3 cm^−1^) and 4T1.2 (22.72±2.5 cm^−1^) cell lines. The data for each cell line included in Table 1 are outlined in the Supplementary Figures (see Supplementary Figures S1–S4).

### Mineralisation of 4T1 and 4T1.2 cells also occurs *in vivo*

**Figure 2:**
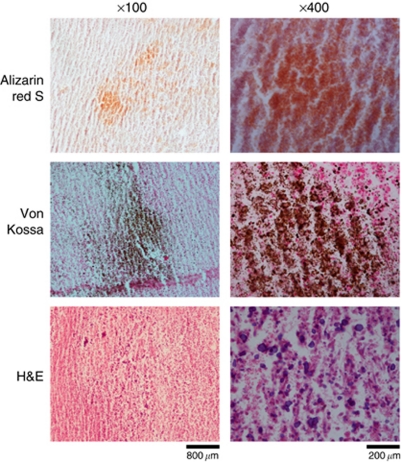
Mineralisation of mouse mammary tumours. Serial sections of 4T1 and 4T1.2 mammary fat pad tumours were stained using alizarin red S, von Kossa (including nuclear fast red counterstain) and H&E. Mineralisation was detected in five out of six primary 4T1.2 tumours and one out of five primary 4T1 tumours. Representative images of 4T1.2 primary tumours are shown. Scale bars represent 800 *μ*m at × 100 magnification and 200 *μ*m at × 400 magnification. Alizarin red S and von Kossa staining are positive for calcium (red) and calcium phosphate (black/brown), respectively. Haematoxylin also stained more intensely in areas of calcifications.

Primary mammary tumours were produced by implanting 4T1 or 4T1.2 cells into the mammary fat pads of female BALB/c mice. Serial sections of these tumours were stained with alizarin red S, von Kossa (with nuclear fast red counterstain) and H&E to assess mineralisation. One of the five 4T1 mammary tumours contained calcifications and mineralisation was detected in five out of six 4T1.2 mammary tumours. A representative mineralising 4T1.2 mammary tumour is shown in Figure 2. Positive staining for calcium (red; alizarin red S) and calcium phosphate (black/brown; von Kossa) was consistently observed in the same region of the tumours between serial sections. Haematoxylin (purple) also stained more intensely in the areas of calcifications.

### Phosphate transport is required for mineralisation of 4T1 cells

**Figure 3:**
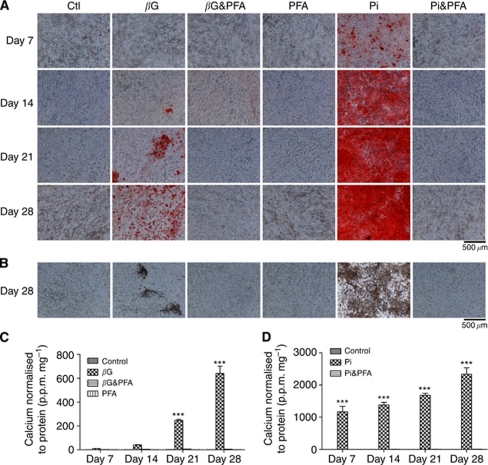
The effect of 1 mM PFA, a known inhibitor of Na-Pi co-transporters, on mineralisation of 4T1 cells. All images are viewed under the light microscope at × 100 magnification and the scale bars represent 500 *μ*m (*n*=3). (**A**) Alizarin red S staining of 4T1 cells treated with 1 mM PFA. (**B**) Von Kossa staining of 4T1 cells treated with 1 mM PFA for 28 days. The effect of 1 mM PFA on mineralisation of 4T1 cells as determined by the o-cresolphthalein calcium assay is shown in (**C**) and (**D**). Each point represents the mean amount of calcium measured in p.p.m. normalised to protein measured in mg, ±s.e.m., two-way ANOVA. ^***^P<0.001 *β*G and Pi *vs* all other treatment groups at each time point. Ctl (control)=regular growth media. *β*G=10 mM *β*G. PFA=1 mM PFA. *β*G&PFA=10 mM *β*G and 1 mM PFA. Pi=10 mM Pi. Pi&PFA=10 mM Pi and 1 mM PFA.

Phosphonoformic acid, a known inhibitor of the type II family of sodium–phosphate cotransporters (Murer *et al*, 2004), was used to investigate the role of phosphate transport in *β*G and Pi-induced mineralisation of 4T1 cells. 4T1 cells stained positive for calcium after treatment with either *β*G or Pi beginning on days 14 and 7, respectively (Figure 3A). However, the addition of PFA in combination with either form of phosphate (*β*G&PFA or Pi&PFA) inhibited mineralisation for up to 28 days. These results were confirmed by von Kossa staining, as shown by representative day 28 images (Figure 3B) and also by a quantitative calcium assay. *β*-Glycerophosphate and Pi treatments resulted in a statistically significant increase in calcium levels beginning on days 21 and 7, respectively, (*P*<0.001; Figures 3C and D). In contrast, the calcium levels of the *β*G&PFA and Pi&PFA groups remained comparable to control levels over time.

### ALP is required for mineralisation of 4T1 cells

**Figure 4:**
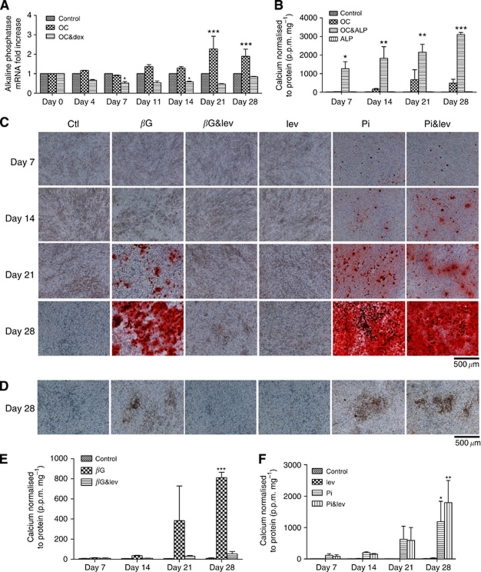
The role of ALP in 4T1 cell mineralisation. (**A**) The expression of ALP mRNA analysed using real-time RT–PCR. The results are expressed in arbitrary units and normalised to the controls at each time point. Each point represents the mean±s.e.m., *n*=3, two-way ANOVA. ^*^P<0.05 OC&dex *vs* control on days 7 and 14. ^***^P<0.001 OC *vs* control and OC&dex on days 21 and 28. (**B**) The effect of exogenous ALP, as determined by the o-cresolphthalein calcium assay. Each point represents the mean amount of calcium measured in p.p.m. normalised to protein measured in mg, ±s.e.m., two-way ANOVA. ^*^P<0.05 OC&ALP *vs* all groups on day 7, ^**^P<0.01 OC&ALP *vs* all groups on days 14 and 21, ^***^P<0.001 OC&ALP *vs* all groups on day 28. (**C**) Alizarin red S staining of 4T1 cells treated with 100 *μ*M levamisole (lev) as viewed under the light microscope at × 100 magnification. The scale bar represents 500 *μ*m. (**D**) Von Kossa staining of 4T1 cells treated with 100 *μ*M lev for 28 days as viewed under the light microscope at × 100 magnification. The scale bar represents 500 *μ*m. *β*G, Pi and Pi&lev samples stain positive for calcium phosphate (black) by day 28. No positive staining was observed in the control, *β*G&lev and lev groups. The effect of lev on mineralisation of 4T1 cells as determined by the o-cresolphthalein calcium assays is shown in (**E**) and (**F**). Each point represents the mean amount of calcium measured in p.p.m. normalised to protein measured in mg, ±s.e.m., two-way ANOVA. ^*^P<0.05 Pi *vs* control on day 28, ^**^P<0.01 Pi&lev *vs* control on day 28, ^***^P<0.001 *β*G *vs* control and *β*G&lev on day 28. Osteogenic cocktail=50 *μ*g ml^–1^ ascorbic acid and 10 mM *β*G. OC&dex=OC including 10^−7^ M dexamethasone. ALP=1 U ml^–1^ ALP. OC&ALP=OC including 1 U ml^–1^ ALP. *β*G=10 mM *β*G. *β*G&lev=10 mM *β*G and 100 *μ*M lev. Lev=100 *μ*M lev. Pi=10 mM Pi. Pi&lev=10 mM Pi and 100 *μ*M lev.

The role of ALP was initially investigated using real-time RT–PCR (Figure 4A). An increase in ALP mRNA expression was detected on days 21 and 28 in mineralising OC-treated 4T1 cells (*P*<0.001). In contrast, there was a general trend for decreased expression of ALP mRNA in the mineralisation impaired OC&dex-treated 4T1 cells, which was statistically significant on days 7 and 14 (*P*<0.05). The effect of exogenous ALP was also investigated. It was found that when ALP was added to the OC, calcium levels were consistently increased compared with the OC group on days 7 (*P*<0.05), 14 (*P*<0.01), 21 (*P*<0.01) and 28 (*P*<0.001; Figure 4B). Next, the effect of levamisole, a known inhibitor of ALP, was investigated in 4T1 cells. *β*-Glycerophosphate and Pi treatment resulted in positive alizarin red S staining beginning on days 14 and 7, respectively, (Figure 4C). The addition of levamisole to *β*G (*β*G&lev) inhibited alizarin red S staining for up to 28 days. In contrast, levamisole had no effect on Pi-induced mineralisation, as shown by positive alizarin red S staining beginning on day 7. These findings were confirmed by von Kossa staining, as shown by representative day 28 images (Figure 4D). These results were also confirmed by quantitative calcium assays. Although an increase in calcium levels was detectable in the *β*G-treated 4T1 cells over time (*P*<0.001 *vs* control and *β*G&lev), no increase was detected in the *β*G&lev group for up to 28 days (Figure 4E). Calcium levels increased over time in the Pi-treated 4T1 cells (*P*<0.05 *vs* control) and similar levels were detected in the Pi&lev group (*P*<0.01 *vs* control). No statistical difference was detected between the Pi and Pi&lev groups at any time point examined for up to 28 days (*P*>0.05; Figure 4F).

### The effect of inhibitors of physiological mineralisation on 4T1 mineralisation

**Figure 5:**
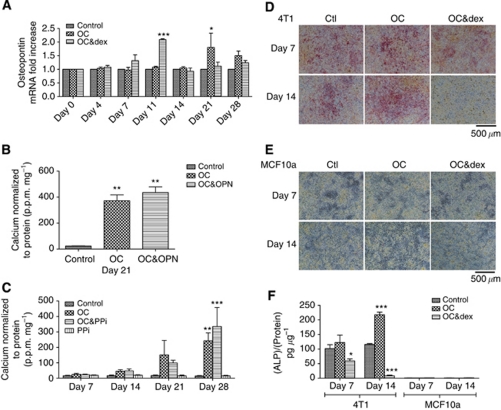
The effect of known inhibitors of physiological mineralisation on 4T1 cells and the influence of endogenous ALP activity. (**A**) The expression of OPN mRNA analysed using real-time RT–PCR. The results are expressed in arbitrary units and normalised to the controls at each time point. Each point represents the mean±s.e.m., *n*=3, two-way ANOVA. ^*^P<0.05 OC *vs* control and OC&dex on day 21. ^***^P<0.001 OC&dex *vs* control and OC on day 11. (**B**) The effect of exogenous OPN on mineralisation of 4T1 cells, as determined by the o-cresolphthalein calcium assay. Each point represents the mean amount of calcium measured in p.p.m. normalised to protein measured in mg, ±s.e.m., one-way ANOVA. ^**^P<0.01 OC and OC&OPN *vs* control on day 21. (**C**) The effect of PPi on mineralisation of 4T1 cells, as determined by the o-cresolphthalein calcium assay. Each point represents the mean amount of calcium measured in p.p.m. normalised to protein measured in mg, ±s.e.m., two-way ANOVA. ^**^P<0.01 OC *vs* control and PPi on day 28, ^***^P<0.001 OC&PPi *vs* control and PPi on day 28. (**D**) An ALP stain carried out on 4T1 cells, as observed under the light microscope at × 100 magnification. The scale bar represents 500 *μ*m. Positive staining for ALP (pink) is observed on days 7 and 14 in the control (Ctl), OC and OC&dex groups. (**E**) An ALP stain carried out on MCF10a cells, as observed under the light microscope at × 100 magnification. The scale bar represents 500 *μ*m. No positive staining for ALP was observed for any treatment group at any time point. (**F**) A comparison of ALP activity in 4T1 and MCF10a cells. The results are expressed in ALP (pg) normalised to protein (*μ*g). Each point represents the mean±s.e.m., two-way ANOVA. ^*^P<0.05 OC&dex *vs* control on day 7 in 4T1 cells, ^***^P<0.001 OC and OC&dex *vs* control on day 14 in 4T1 cells. Osteogenic cocktail=50 *μ*g ml^–1^ ascorbic acid and 10 mM *β*G. OC&dex=OC including 10^−7^ M dexamethasone. OC&PPi=OC and 3.5 *μ*M pyrophosphate. PPi=3.5 *μ*M pyrophosphate. OC&OPN=OC and 0.5 *μ*g ml^–1^ OPN.

The role of OPN was investigated using real-time RT–PCR (Figure 5A). An increase in OPN mRNA expression was detected on day 21 in mineralising OC-treated 4T1 cells (*P*<0.05) and on day 11 in the mineralisation impaired OC&dex group (*P*<0.001). The effect of exogenous OPN was also investigated in mineralising 4T1 cells using a quantitative calcium assay (Figure 5B), however, the addition of 0.5 *μ*g ml^–1^ OPN to the OC (OC&OPN) resulted in levels of calcium similar to the OC group by day 21 and no differences were detected between these two groups (*P*>0.05). Similarly the exogenous addition of PPi, a known inhibitor of physiological mineralisation, was found to have no effect on 4T1 cells (Figure 5C). An increase in calcium was detected in 4T1 cells treated with the OC and 3.5 *μ*M PPi (OC&PPi) compared with the control group by day 28 (*P*<0.001; Figure 5C). These levels were comparable to the elevated calcium levels of the OC group at all-time points and no differences were detected between these two groups (*P*>0.05).

### High endogenous ALP activity correlates with mineralisation potential

Having found that exogenous PPi and OPN do not inhibit mineralisation of 4T1 cells as expected, the endogenous levels of ALP were investigated as a potential cause. An ALP stain was used to visualise ALP activity in 4T1 cells grown for up to 14 days. A pink colour indicates positive staining for ALP and representative images are shown in Figure 5D. Positive staining was observed in the control samples on days 7 and 14. A further increase in staining was detected in the OC group, whereas a decrease in ALP staining was observed in the OC&dex group at both time points. Alkaline phosphatase staining was also carried out on the MCF10a cell line (Figure 5E), which was previously established as non-mineralising under the same experimental conditions (Table 1). No positive staining for ALP was observed for any treatment group for up to 14 days in the MCF10a cells. These results were also confirmed by a quantitative ALP assay (Figure 5F). By day 14, an increase in ALP activity was found in the OC group compared with the control group for the 4T1 cell line (*P*<0.001). In contrast, a decrease in ALP activity was detected in the OC&dex group on days 7 (*P*<0.05) and 14 (*P*<0.001). In direct comparison, the MCF10a cell line had little or no ALP activity for any treatment group for up to 14 days.

### *β*G, calcium and exogenous HA increase tumour cell migration

**Figure 6:**
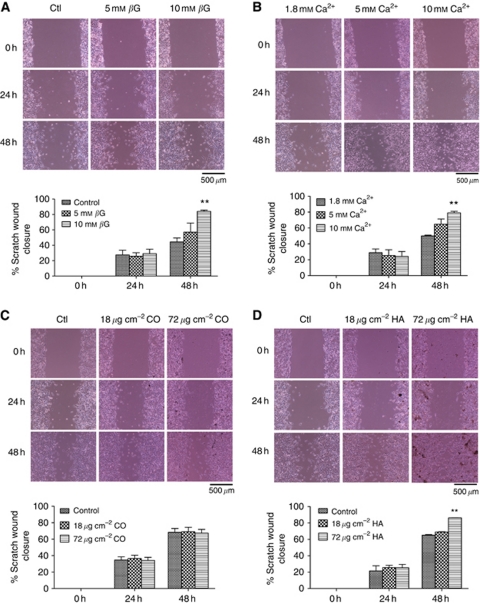
Investigating the effect of increasing concentrations of *β*G, calcium (Ca^2+^), CO and HA on 4T1 cell migration using a scratch wound assay. (**A**) The scratch wound assay of 4T1 cells treated with *β*G. By 48 h, an increase in cell migration is observed with 10 mM *β*G treatment compared with the control group (ctl; growth media containing 0.5% FBS), which was found to be statistically significant when quantified using Scion image software. (**B**) The scratch wound assay of 4T1 cells treated with increasing concentrations of calcium. By 48 h, an increase in cell migration is observed with 10 mM Ca^2+^ treatment compared with the 1.8 mM Ca^2+^ group (growth media containing 0.5% FBS), which was found to be statistically significant when quantified using Scion image software. (**C**) The scratch wound assay of 4T1 cells treated with increasing concentrations of CO. No significant differences in cell migration were detected between the different treatment groups at any time point. (**D**) The scratch wound assay of 4T1 cells treated with increasing concentrations of HA. An increase in cell migration is observed for the 72 *μ*g cm^–2^ HA-treated cells compared with the control group by 48 h, which was confirmed as statistically significant when quantified using Scion image software. All images are viewed under a light microscope at × 100 magnification at 0, 24 and 48 h (*n*=3). The scale bars represent 500 *μ*m. For quantification, each point represents the mean percentage scratch wound closure ±s.e.m., two-way ANOVA. ^**^P<0.01 10 mM *β*G, 10 mM Ca^2+^ and 72 *μ*g cm^–2^ HA *vs* control at 48 h.

Scratch wounds were made in monolayers of 4T1 cells and cell migration was recorded in response to increasing concentrations of *β*G, calcium (Ca^2+^), CO and HA. It was found that an increasing number of cells migrated into the scratch areas over time for all treatment groups. However, larger numbers of cells were observed in the 10 mM *β*G, 10 mM Ca^2+^ and 72 *μ*g cm^–2^ HA groups compared with the control groups by 48 h (Figures 6A, B and D, respectively). These results were found to be statistically significant by quantitative analysis of wound closure using Scion image software (*P*<0.01). In contrast, no significant changes were detected for CO treatment for either 18 *μ*g cm^–2^ or 72 *μ*g cm^–2^ by 48 h (*P*>0.05 *vs* control; Figure 6C).

## Discussion

Mammary microcalcifications are one of the most reliable mammographic features of breast cancer and are often the sole indicator of breast disease in its nonpalpable form. A recent study has shown that the carbonate content of mammary microcalcifications composed of HA may be associated with lesion grade (Baker *et al*, 2010). However, despite their value for the early detection of breast cancer and potential prognostic importance, the mechanisms by which mammary microcalcifications form are unclear at best. It has remained uncertain whether they are produced by an active cellular process or if calcifications are a sign of cellular degeneration.

We have demonstrated here for the first time that mammary cell lines are capable of mineralising *in vitro*. This has been shown in detail for the mouse metastatic 4T1 cell line, which was found to mineralise by day 11 when treated with an OC. Raman microspectroscopy was used to identify the calcium species being deposited by the 4T1 cells. The Raman band observed at 960 cm^−1^ is indicative of the presence of HA, which is a clinically relevant species of calcium that is associated with both benign and malignant breast conditions (Büsing *et al*, 1981; Radi, 1989; Haka *et al*, 2002).

Mineralisation of additional mammary cell lines in the presence of an OC was also confirmed, which included the metastastic mouse mammary 4T1.2 cell line and the highly invasive human mammary Hs578Ts(i)_8_ cell line. In contrast, the comparatively less invasive Hs578T cells and the non-tumourigenic MCF10a cells were not capable of mineralising under the same conditions. This suggests that mineralisation potential in this *in vitro* model is associated with mammary cell phenotype, as only the tumourigenic mammary cells were capable of producing HA. In addition, a narrower FWHH was found for the HA deposited by the Hs578Ts(i)_8_ cells (15.9±1.7 cm^−1^) compared with all other mineralising cell lines. Narrowing of the 960 cm^−1^ phosphate stretching peak has been associated with lower carbonate content (Haka *et al*, 2002) and lower carbonate content with increasing tumour grade (Baker *et al*, 2010), reflecting the highly invasive nature of the Hs578Ts(i)_8_ cells. Haka *et al* (2002) previously reported that type II microcalcifications occurring in benign breast lesions had an average FWHH of 18.0±0.5 cm^−1^, whereas deposits in lesions diagnosed as DCIS had an average FWHH of 17.0±0.5 cm^−1^. FWHH data for mineral resulting from murine sources have not previously been reported but both mouse cell lines (4T1 and 4T1.2) generated FWHH significantly higher (21.7±1.2–23.9±3.0 cm^−1^) than the human Hs578Ts(i)_8_ cell line or previously reported data for human breast biopsies.

Osteogenic media was used throughout the *in vitro* experiments because the addition of exogenous phosphate to investigate mineralisation potential is routine in mineralisation studies of numerous other pathologies, including osteoblasts and vascular smooth muscle cells *in vitro* (Shioi *et al*, 1995; Coelho and Fernandes, 2000). In the human body, phosphate is the most abundant intracellular anion most commonly found in the form of adenosine phosphates, (AMP, ADP and ATP) and in DNA and RNA and can be released by the hydrolysis of ATP or ADP. Consequently, it is not unreasonable to assume that in the early tumour niche, rapidly proliferating populations of cells may be exposed to physiologically localised high levels of phosphate. The results of a small *in vivo* study are presented here, showing that 4T1 and 4T1.2 cells grown in the mammary fat pad of BALB/c mice are capable of producing calcifications without any experimental alteration to phosphate levels. The time required for mineralisation of the 4T1 and 4T1.2 cells *in vitro* was relatively similar (11 *vs* 14 days, respectively), however, the detection of mineralisation in the mammary fat pads *in vivo* differed, with microcalcifications more common in the 4T1.2 tumours. The exact reasons for this are unclear but may be related to the cells phenotype because the 4T1.2 cell line is a variant isolated from parental 4T1 cells through single cell cloning and is known to develop overt metastases in bone, lung and lymph nodes (Lelekakis *et al*, 1999). Unlike the 4T1 cell lines, the 4T1.2 cell lines have a high propensity to spread to the auxiliary lymph nodes with metastasis thought to occur through a lymphogenous route (Eckhardt *et al*, 2005). Their increased tendency to mineralise may be another feature of their aggressive phenotype. Never the less both cell lines 4T1 and 4T1.2 are capable of mineralising *in vivo* in the absence of any exogenous factors or experimental alternation to physiological phosphate levels. This demonstrates an innate ability to produce mammary microcalcifications during the course of tumour development. This finding also supports our *in vitro* work and demonstrates that mineralisation is not simply due to the presence of cell culture medium constituents or a phenomenon resulting from prolonged monolayer culture.

Dexamethasone, a synthetic glucocorticoid, was also investigated as it has been shown to enhance the mineralisation of certain bone-like cells (Maniatopoulos *et al*, 1988; Coelho and Fernandes, 2000). As the mineralisation potential of mammary cells *in vitro* was previously unexplored, two versions of the OC (with and without dexamethasone) were investigated in order to determine the optimum conditions for mineralisation. We have shown that dexamethasone inhibits mineralisation of both the mouse 4T1 and 4T1.2 cell lines. In contrast, the presence of dexamethasone in the OC is required for the mineralisation of the human Hs578Ts(i)_8_ cell line. This species-specific effect has also been observed in studies of *in vitro* mineralisation using osteoblasts. Dexamethasone enhances mineralisation of human bone marrow cells (Coelho and Fernandes, 2000), whereas dexamethasone inhibits mineralisation of the mouse osteoblast MC3T3-E1 cell line *in vitro* (Lian *et al*, 1997). It has been suggested that the differing effect of dexamethasone may depend on the stage of osteoblast maturation of the cell lines investigated (Lian *et al*, 1997). In addition, the reason for the species-specific effect of dexamethasone may be due to differences in the metabolism of dexamethasone between human and mouse cells, which results in differing major metabolites (Tomlinson *et al*, 1997). These different metabolites could potentially have varying effects on cell signalling and gene transcription associated with mineralisation.

Using the established *in vitro* model of mammary mineralisation, the molecular mechanisms involved in this process were investigated. The role of phosphate transport was studied using PFA, a known inhibitor of the type II family of sodium-phosphate (Na-Pi) cotransporters (Murer *et al*, 2004). Inhibition of these Na-Pi cotransporters using PFA completely abolished both *β*G-induced and Pi-induced mineralisation of 4T1 cells. This indicates that, regardless of the source, internalisation of phosphate is essential for 4T1 mineralisation to occur in this model. The transport of phosphate across a membrane by a phosphate pump suggests that an active regulated process is at work and that the mineralisation observed is not simply due to precipitation within the media or the result of necrotic debris. In addition, it has been reported that the expression of the NaPi-IIb cotransporter is elevated in breast cancer tissue in comparison with normal tissue (Chen *et al*, 2010). No other researchers have investigated the involvement of these transporter pumps in mammary cell mineralisation, however, upregulation of Na-Pi cotransporters in mammary epithelial cells by disease state may facilitate localised mineralisation even when serum phosphate levels are in the normal physiological range.

The role of ALP was also investigated, as this enzyme has a well-documented role in physiological HA production (Owen *et al*, 1990; Orimo, 2010). An increase in ALP mRNA was detected in the mineralising OC-treated 4T1 cells and the exogenous addition of ALP was found to enhance 4T1 cell mineralisation. It was also found that levamisole, which is a known inhibitor of ALP, inhibits *β*G-induced mineralisation, but not Pi-induced mineralisation of 4T1 cells. This suggests that ALP may have a similar function in mineralising mammary cell lines as observed for osteoblasts. Alkaline phosphatase is thought to hydrolyse organic phosphate, liberating Pi, which can be used by cells to create HA (Robison, 1923; Simao *et al*, 2007). When ALP function is inhibited by levamisole treatment of the 4T1 cells, no *β*G-induced mineralisation takes place. This is likely due to the impaired production of Pi from *β*G. In contrast, impaired ALP function has no impact on Pi-induced mineralisation, as Pi is already freely available to be used by the cells for HA production. This would also explain why mineralisation is consistently detected at earlier time points for the Pi-treated cells, as hydrolysis of *β*G is thought to be time dependent.

The potential role of OPN was investigated in our *in vitro* model of mammary cell mineralisation, as this phosphoprotein is highly expressed in bone and is known to be upregulated during bone formation (Chen *et al*, 1992; Standal *et al*, 2004). Increased expression of OPN has also been reported in breast tumours containing microcalcifications (Bellahcene and Castronovo, 1995). The endogenous expression of OPN mRNA was found to be upregulated in the mineralising OC group on day 21. This provides further evidence that mammary cells mineralise in a regulated manner similar to osteoblasts. In addition, an increase in OPN mRNA was detected on day 11 in the OC&dex group. This may contribute to the diminished levels of mineralisation observed in the OC&dex group compared with the OC. Osteopontin is thought to negatively regulate mineralisation by binding to and preventing further HA crystal growth (Boskey *et al*, 1993; Gericke *et al*, 2005; Jahnen-Dechent *et al*, 2008). The relatively early increased expression of OPN mRNA coupled with the trend for decreased ALP mRNA expression in the OC&dex group may explain why less mineralisation was detected in this treatment group.

The role of OPN was investigated further by examining the effect of exogenous OPN on 4T1 mineralisation, as this is known to inhibit mineralisation of osteoblasts *in vitro* (Boskey *et al*, 1993; Addison *et al*, 2007). However, the exogenous addition of OPN had no effect on 4T1 cell mineralisation when added to the OC. Similarly, exogenous treatment with PPi, another known inhibitor of osteoblast mineralisation (Addison *et al*, 2007), had no effect on 4T1 mineralisation. These results indicate that the process of mammary mineralisation may not be completely identical to that of physiological mineralisation. We hypothesised that this observation may be due to high endogenous level of ALP, as ALP has been shown to remove the inhibitory effect of both OPN and pyrophosphate through dephosphorylation and hydrolysis, respectively. Osteopontin is only active in its phosphorylated form (Boskey *et al*, 1993; Hunter *et al*, 1994; Keykhosravani *et al*, 2005) and pyrophosphate can be hydrolysed to Pi, which removes its inhibitory effect (Lomashvili *et al*, 2004; Addison *et al*, 2007; Simao *et al*, 2007). Therefore, an ALP stain and assay were used to examine the endogenous ALP activity in the mineralising 4T1 cell line compared with the non-mineralising MCF10a cell line. As predicted, comparatively high levels of ALP activity were detectable even in the control 4T1 cells, whereas little or no ALP activity was detectable in the MCF10a cell line for any treatment investigated. Additional increases in ALP activity were observed in the 4T1 cells in response to the OC. Therefore, enhanced expression of already high endogenous levels of ALP could remove the inhibitory effects of OPN and pyrophosphate in this *in vitro* model.

The effect of the *in vitro* culture conditions that resulted in mineralisation were also investigated in relation to cell migration. Using scratch wound assays it was found that 10 mM *β*G enhanced migration of the 4T1 cells by 40% after 48 h of treatment compared with the control group. As microcalcifications may form in response to locally high concentrations of calcium, increasing concentrations of calcium were also examined. In all, 10 mM calcium was found to increase migration of 4T1 cells by ∼30% after 48-h treatment in comparison with the control group. These data suggest that the microenvironment or niche conditions preceding, and ultimately leading to, mammary mineralisation may promote migration of 4T1 cells. Next, the functional effects of exogenous crystallised CO and HA on 4T1 cell migration were investigated. It was found that CO crystals of up to 72 *μ*g cm^–2^ have no effect on cell migration for up to 48 h of treatment. In contrast, after 48 h of treatment with 72 *μ*g cm^–2^ of HA crystals, 4T1 cell migration increased by 20% in comparison with the control group. As increasing concentrations of phosphate or calcium had slightly more pronounced migratory effects than the addition of exogenous crystallised HA, it is possible that the pre-mineralisation phase is of most biological significance. Mineralisation will result in sequestering of calcium and phosphate, which may be chronically released and its effects may be more pronounced in an assay carried out over a much longer time period. The increase in cell migration observed for HA treatment, but not seen with CO, lends weight to the theory that HA and the conditions, which precede its deposition may have functional biological consequences within the tumour microenvironment. These findings suggest that deposited HA may be capable of stimulating migration of the surrounding cells within the tumour microenvironment, which could contribute to breast cancer progression. We have previously reported that HA treatment of several mammary cell lines upregulates the production of MMPs (Morgan *et al*, 2001), which are known to contribute to breast cancer invasion of surrounding tissue (Lochter and Bissell, 1999). Therefore, the presence of HA within the tumour microenvironment could contribute to breast cancer progression and ultimately metastasis by producing MMPs to degrade the basement membrane and enhance the migration of cells to the surroundings tissues.

**Figure 7:**
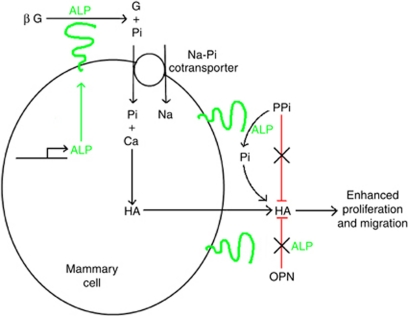
A proposed mechanism of mammary cell mineralisation. *β*G is hydrolysed to glycerol (G) and Pi by ALP. Inorganic phosphate is internalised by the type II family of Na-Pi cotransporters. Once inside the cell, Pi combines with calcium (Ca) to form HA. Upregulation of ALP mRNA takes place. Hydroxyapatite enters the extracellular matrix, by an as of yet unknown mechanism. Inorganic pyrophosphate acts as a natural inhibitor of HA formation. However, the overexpression of ALP by tumourigenic mammary cells may result in PPi hydrolysis to Pi, which can subsequently be incorporated into HA. Osteopontin is also a natural inhibitor of HA formation, however, overexpression of ALP may dephosphorylate OPN rendering it inactive and thereby removing its inhibitory effect. Hydroxyapatite crystals present in the extracellular matrix enhance the proliferation and migration of surrounding cells and further aggravate tumour growth and metastasis.

Based on the results discussed in this paper, we put forward a mechanism for *in vitro* 4T1 mammary cell mineralisation (Figure 7). We suggest that ALP on the surface of mammary cells hydrolyses *β*G to glycerol and Pi. The Pi produced may then be transported into the mammary cells by the type II family of Na-Pi cotransporters. Once inside the cell, the Pi could combine with any available sources of calcium to produce HA crystals. Mammary cells have an innate ability to concentrate calcium ions, as this occurs naturally to allow lactation to take place (Virk *et al*, 1985; VanHouten *et al*, 2007). Hydroxyapatite would then leave the cells by an as of yet unknown mechanism. Osteopontin and PPi within the extracellular matrix have no effect on limiting crystal growth, possibly due to the upregulation of ALP. Alkaline phosphatase may act by dephosphorylation of OPN and hydrolysing PPi to Pi, which could be subsequently incorporated in growing HA crystals. Therefore, we propose that an imbalance of these regulators of physiological mineralisation within the tumour microenvironment may be responsible for the formation of pathological mammary microcalcifications. Hydroxyapatite and the biochemical conditions, which initiate its deposition could then enhance the migration of the surrounding mammary cells. In addition, the osteomimicry capabilities of the mammary cells may also contribute to their ability to metastasise to bone once in the circulation, as they would possess an innate ability to survive within the bone microenvironment.

One of the models most notable characteristic is that the cells generate HA, a calcium species, which is found in human clinical samples. Under the conditions examined *in vitro* only the more tumourigenic cell lines were capable of mineralising, suggesting that our *in vitro* model and the proposed mechanism may be most useful for the study of calcium deposition associated with malignancy of the breast. As with all *in vitro* models there are limitations in the recapitulation of the complex interactions, which may take place in an *in vivo* scenario. Despite this our cell line model has the advantage of providing an unlimited source of homogenous material, and is free of potential contaminating stromal cells, offering the opportunity to perform reproducible mechanistic experiments in a controlled environment. Although the transposability to tumours of results obtained from cell lines universally remains a matter of debate this model represents a starting point in an area, which was previously unexplored.

This work offers insights into the process of malignant mineralisation in breast tissue and will ultimately pave the way for a more complete understanding of the complex relationships between the mineral, the microenvironment and the differentiation state of the cells, which deposit them. As more breast cancer cases are being detected in their pre-palpable stage, largely through the presence of mammary microcalcifications, it is imperative that current research efforts reflect these developments and focus on understanding the biology underlying one of the most reliable markers of preclinical breast cancer.

## Figures and Tables

**Table 1:** Summary of the mineralisation potential of a panel of mammary cell lines as assessed by alizarin red S, von Kossa staining, quantitative calcium assay and Raman microspectroscopy

**Cell line**	**Cell phenotype**	**Mineralise *in vitro***	**Initial histological staining**	**Conditions for mineralisation**	**Raman shift (cm^−1^)**	**FWHH (cm^−1^)**	**Mineral species**
MCF10a	Human, immortalised, normal breast epithelial from benign breast tissue of a woman with fibrocystic disease	No	NA	NA	NA	NA	NA
Hs578T	Human epithelial, mammary carcinoma of 74-year-old Caucasian woman	No	NA	NA	NA	NA	NA
Hs578Ts(i)_8_	A more invasive subclone of the Hs578T cells	Yes	Day 21	OC&dex	961.10±0.5	15.85±1.7	HA
4T1	Mouse, lung metastasising subclone from a spontaneous mammary tumour	Yes	Day 11	OC	960.84±0.7	22.45±3.4	HA
				OC&dex	959.55±0.9	23.89±3.0	HA
4T1.2	Mouse, metastasising subclone isolated from the 4T1	Yes	Day 14	OC	961.47±0.6	21.67±1.2	HA
	cell line			OC&dex	961.56±0.5	22.72±2.5	HA

Abbreviations: FWHH=full width at half height as determined by Raman spectroscopy; HA=hydroxyapatite; NA=not applicable; OC (osteogenic cocktail)=50 *μ*g ml^–1^ ascorbic acid and 10 mM *β*-glycerophosphate; OC&dex=OC including 10^−7^ M dexamethasone.
