# Supplementary material for: Microcalcifications in breast cancer: novel insights into the molecular mechanism and functional consequence of mammary mineralisation
Source: Br J Cancer. 2012 Jan 10;106(3):525–37. doi: 10.1038/bjc.2011.583 (PMC3273345; doi:10.1038/bjc.2011.583)
Supplement: Supplementary Figure S1 [file bjc2011583x1.doc]

**A B**

500µm 500µm

**C**
