# Supplementary material for: Microcalcifications in breast cancer: novel insights into the molecular mechanism and functional consequence of mammary mineralisation
Source: Br J Cancer. 2012 Jan 10;106(3):525–37. doi: 10.1038/bjc.2011.583 (PMC3273345; doi:10.1038/bjc.2011.583)
Supplement: Supplementary Figure Legends [file bjc2011583x5.doc]

**Supplemental figure legends**

**Supplemental figure 1.**

Investigating the mineralization potential of non-tumorigenic human mammary MCF10a cells. Alizarin red S (A) and von Kossa (B) staining as viewed under a light microscope at 100x magnification. The scale bars represent 500µm. No positive staining for either calcium (red) or calcium phosphate (black/brown) was detected for up to 28 days, regardless of treatment. (C) The calcium content of MCF10a cells as determined by the o-cresolphthalein calcium assay and normalized to protein. No changes in calcium were detected between the treatment groups compared to the control group over time. Each point represents the mean amount of calcium measured in ppm normalized to protein measured in mg, +/- SEM, (n=3). Control = regular growth media. OC (osteogenic cocktail) = regular growth media supplemented with 50µg/ml ascorbic acid and 10mM β-glycerophosphate. OC&dex = regular growth media supplemented with 50µg/ml ascorbic acid, 10mM β-glycerophosphate and 10^-7^M dexamethasone.

**Supplemental figure 2.**

Investigating the mineralization potential of the invasive human mammary Hs578T cell line. Alizarin red S (A) and von Kossa (B) staining as viewed under a light microscope at 100x magnification. The scale bars represent 500µm. No positive staining for either calcium (red) or calcium phosphate (black/brown) was detected for up to 28 days, regardless of treatment. (C) The calcium content of Hs578T cells as determined by the o-cresolphthalein calcium assay. No significant changes in calcium were detected between the treatment groups compared to the control group at any time point. A slight trend for increased calcium was observed in the OC&dex group on day 28, however this did not reach statistical significance. Each point represents the mean amount of calcium measured in ppm normalized to protein measured in mg, +/- SEM, two-way ANOVA (n=3). OC (osteogenic cocktail) = 50µg/ml ascorbic acid and 10mM β-glycerophosphate. OC&dex = OC and 10^-7^M dexamethasone.

**Supplemental figure 3.**

Investigating the mineralization potential of the highly invasive human mammary Hs578Ts(i)_8_ subclone. Alizarin red S (A) and von Kossa (B) staining as viewed under a light microscope at 100x magnification. The scale bars represent 500µm. Positive staining for calcium (red) and calcium phosphate (black/brown) was observed in the OC&dex group beginning on day 21 and increasing in intensity by day 28. (C) The calcium content of Hs578Ts(i)_8_ cells as determined by the o-cresolphthalein calcium assay. The calcium content of the OC&dex group was significantly increased compared to the control and OC groups beginning on day 21 and continuing to day 28. Each point represents the mean amount of calcium measured in ppm normalized to protein measured in mg, +/- SEM, two-way ANOVA (n=3). ***P<0.001 OC&dex vs. control and OC on days 21 and 28. (D) Raman spectroscopy of mineralizing Hs578Ts(i)_8_ cells grown in the OC&dex for 28 days. The Raman spectrum contains a peak at 960cm^-1^, as indicated by the arrow. Peaks at 620cm^-1^, 1000cm^-1^ and 1040cm^-1^ (*) were generated by the polystyrene surface of the 6-well plates. OC (osteogenic cocktail) = 50µg/ml ascorbic acid and 10mM β-glycerophosphate. OC&dex = OC and 10^-7^M dexamethasone.

**Supplemental figure 4.**

Investigating the mineralization potential of metastatic mouse mammary 4T1.2 cells. Alizarin red S (A) and von Kossa (B) staining as viewed under a light microscope at 100x magnification. The scale bars represent 500µm. Both the OC and the OC&dex groups began to stain positive for calcium (red) and calcium phosphate (black/brown) on day 14. This positive staining increased in intensity over time with the strongest staining for these samples on day 28 in the OC group. (C) The calcium content of 4T1.2 cells as determined by the o-cresolphthalein calcium assay. Calcium content is significantly increased in the OC group on days 21 and 28. Calcium content also increased in the OC&dex group over time, however this did not reach statistical significance. No changes in calcium levels normalized to protein were observed in the control group over time. Each point represents the mean amount of calcium measured in ppm normalized to protein measured in mg, +/- SEM, two-way ANOVA (n=3). *P<0.05 OC vs. control on day 21, **P<0.01 OC vs. control on day 28. (D) Raman spectroscopy of mineralizing 4T1.2 cells grown in the OC for 28 days. The Raman spectrum contains a peak at 960cm^-1^, as indicated by the arrow. Peaks at 1000cm^-1^ and 1040cm^-1^ (*) were generated by the polystyrene surface of the 6-well plates. OC (osteogenic cocktail) = 50µg/ml ascorbic acid and 10mM β-glycerophosphate. OC&dex = OC and 10^-7^M dexamethasone.
